# Improving the quality of terminal cleaning & disinfection and reducing nosocomial outbreaks during COVID-19 pandemic in medical center of TAIWAN

**DOI:** 10.1017/ash.2025.86

**Published:** 2025-09-03

**Authors:** FuChieh Chang, Chang-Pan Liu

**Affiliations:** 1 Infection Control Center, MacKay Memorial Hospital, Taipei city, Taiwan; 2 Division of Infectious Diseases, Department of Internal Medicine, MacKay Memorial Hospital, Taipei city, Taiwan

## Abstract

**Introduction:** Severe acute respiratory syndrome coronavirus-2 (SARS-CoV-2) is highly contagious in humans, and in May 2021, an outbreak occurred in the Wanhua district of Taiwan. During the Coronavirus disease 2019 (COVID-19) pandemic, we implemented various rules to prevent the spread of SARS-CoV-2- alpha in the hospital. This included establishing four special wards (dedicated care wards) specifically for patients infected with SARS-CoV-2-alpha. When patients were discharged, we conducted real-time polymerase chain reaction (RT-PCR) testing on the terminal environment to ensure that SARS-CoV-2- alpha was not present. Maintaining a negative test result was crucial for preventing cross-infection and further outbreaks. The goal of this study was to identify effective intervention measures to improve the quality of terminal cleaning and achieving overall infection control in the hospital. **Methods:** After cleaning and disinfection of the dedicated care wards by the cleaning staff as per the recommendations of the Centers for Disease Control (CDC), we collected three swabs from different areas in one ward. We used Roche and GeneXpert instruments for COVID-19 RT-PCR testing. However, because the test results were not ideal, we introduced ultraviolet-C (UV-C) machines and a disinfectant solution containing hydrogen peroxide (H_2_O_2_) into our current cleaning and disinfection procedures. **Results:** The negative test result ratios for RT-PCR testing were 80.13% when cleaning was done with bleach- only method as a disinfectant (without intervening with other methods); 92.81% when intervening with UV-cC machine disinfection for 5 minutes was done before bleach disinfection; 96.19% when bleach was replaced with a disinfectant solution containing H_2_O_2_ and intervening with UV-C disinfection. **Conclusion:** The quality of terminal cleaning and disinfection was a key factor in reducing nosocomial outbreaks. We could consider using UV-C machine and H_2_O_2_ disinfectant to intervene in the current bleach disinfection method to enhance the quality of terminal cleaning and disinfection in the hospital.

